# Detection of melatonin and 5-HTP in dietary supplements based on multiple spectra

**DOI:** 10.3389/fnut.2025.1532092

**Published:** 2025-01-28

**Authors:** Huiyu Yang, Xinrui Zhang, Yang Gao, Zhuang Peng, Bo Su, Kai Li, Cunlin Zhang

**Affiliations:** ^1^Department of Physics, Capital Normal University, Beijing, China; ^2^Beijing Key Laboratory for Terahertz Spectra and Imaging, Beijing, China; ^3^Key Laboratory of Terahertz Optoelectronics, Ministry of Education, Beijing, China; ^4^Department of Chemistry, Capital Normal University, Beijing, China

**Keywords:** terahertz time-domain spectroscopy, melatonin, L-tryptophan, 5-hydroxytryptophan, dietary supplements

## Abstract

**Introduction:**

Melatonin and 5-hydroxytryptophan (5-HTP), known for benefits in regulating sleep and combating depression, respectively, are incorporated into dietary supplements. Rapid and accurate identification of dietary supplement types and their contents remains a significant challenge in ensuring food safety.

**Methods:**

In this study, qualitative and quantitative analysis of melatonin and 5-HTP was performed using Raman spectroscopy, powder X-ray diffraction (PXRD), and terahertz time-domain spectroscopy (THz-TDS). Purity and crystal structures of the samples were investigated using Raman spectroscopy and PXRD, establishing the foundation for terahertz (THz) simulations.

**Results and discussion:**

The Raman spectroscopy results demonstrate that the characteristic Raman peaks of melatonin and 5-HTP in the range from 170 cm^−1^ to 1700 cm^−1^ were observed at 1356 cm^−1^ and 1,304 cm^−1^, respectively. Results of THz revealed that melatonin and 5-HTP each have five THz characteristic peaks, which distinguish these substances. The peak of melatonin at 1.23 THz shows a good linear fit with the mass fraction, while 5-HTP has a similar relationship at 1.14 THz. Then, L-tryptophan, a common contaminant in the production of melatonin and 5-HTP, was successfully identified within the mixture. Finally, it is demonstrated that THz technology can effectively detect melatonin and 5-HTP in commercial dietary supplements. This study establishes a rapid, efficient, and non-destructive approach for the regulation and quantitative analysis of dietary supplements.

## 1 Introduction

Melatonin (N-acetyl-5-methoxytryptamine) is an indoleamine hormone produced in the pineal gland and is widely found in various organisms. It plays multiple roles, including regulating sleep, slowing aging, and showing potential in treating cancer, coronary heart disease, and Alzheimer’s disease (1, 2). Meanwhile, 5-HTP is a crucial precursor for serotonin (3) and melatonin synthesis, demonstrating promise in treating depression (4) and Parkinson’s disease (5). However, its production decreases with age, which has sparked interest in dietary supplements to alleviate age-related degenerative diseases.

In the United States, the Food and Drug Administration (FDA) classifies melatonin and 5-HTP as dietary supplements rather than drugs, leading to significant variability in market doses. Overconsumption of melatonin can lead to side effects such as hypothermia and infertility, while excessive intake of 5-HTP may induce serotonin syndrome (6, 7). Studies have identified contaminants like L-tryptophan in dietary supplements, and excessive intake can potentially lead to eosinophilic myalgia syndrome (EMS) (8–10). EMS is a rare and potentially fatal neurological disorder characterized by severe myalgia and significant peripheral eosinophilia. Initial epidemiological studies, along with a national surveillance program initiated by the Centers for Disease Control and Prevention (CDC) in the United States, have found a strong association between the onset of EMS and the use of L-tryptophan dietary supplements (11). As a result, the use of L-tryptophan (the precursor of 5-HTP) as a dietary supplement has been suspended. Given the potential issues associated with these supplements, we advocate for increased scrutiny and strengthened pharmaceutical regulation of dietary supplements.

Current drug detection methods include liquid chromatography with diode array detection (LC-DAD) (12), RP-C18 HPLC combined with fluorescence detection (13), high-performance liquid chromatography with ultraviolet detection (14), voltammetry (15), and capillary electrophoresis with laser-induced native fluorescence detection (CE/LINF) (16). These methods have been effective in detecting melatonin and 5-HTP. However, traditional liquid phase and mass spectrometry equipment are expensive, have slow detection speeds, and can be destructive to samples. Therefore, there is a pressing need to develop fast and nondestructive detection methods (17). Most food substrates are complex and diverse, making it challenging to find a single technology that meets all the requirements for food detection simultaneously. THz radiation, also known as far-infrared radiation, is a high-frequency electromagnetic wave with a frequency range of 0.1 to 10 THz and a wavelength between 3,000 and 30 μm (18, 19). They are characterized by low energy, strong penetration, rapid detection, and non-invasive properties. Their unique THz fingerprint spectrum allows for the quantitative detection of chemical substances in food (20). This method has been successfully applied to analyze various samples, including pesticides in food matrices like wheat and rice flour (21), L-arginine and *α*-lactose in dietary supplements (22), and bovine serum albumin in thin film/solution (23). Therefore, THz spectroscopy is widely used as a non-destructive and cost-effective method in various fields, including food and agriculture (24, 25). Currently, the application of THz spectroscopy technology in food detection primarily focuses on samples in controlled matrices, with few studies verifying its accuracy in detecting actual food commodities available in the market.

This study examined the Raman spectra of melatonin and 5-HTP to assess their purity. Subsequently, the crystal structures of these compounds were analyzed using PXRD, which provided the foundation for simulating their THz spectra. The focus then shifted to studying the THz spectra of both substances, employing density functional theory (DFT) calculations based on their crystal structures to simulate their spectral characteristics. The comparison between theoretical simulations and experimental results showed a high level of consistency. In THz experiments, we initially detected melatonin and 5-HTP at various mass fractions, demonstrating the THz-TDS system’s capability to differentiate between these samples. Linear fitting was then performed on each absorption peak at different mass fractions, revealing strong fits at 1.23 THz for melatonin and 1.14 THz for 5-HTP, with correlation coefficients (R^2^) of 0.99620 and 0.95995, respectively. Then, L-tryptophan was separately mixed with melatonin and 5-HTP, and detection was conducted using the THz-TDS system. The experimental results highlighted the effectiveness of THz technology in identifying melatonin and 5-HTP contamination. Finally, two commercially available dietary supplements were purchased and analyzed to prove the practicability of the THz experiment. Results revealed that their THz absorption spectra displayed characteristic peaks consistent with those identified in the pure samples. This study establishes the groundwork for quantitatively analyzing components in dietary supplements using THz technology and provides a reliable method for overseeing and testing dietary supplement products in the market.

## 2 Materials and methods

### 2.1 Sample preparation

**Figure 1:**
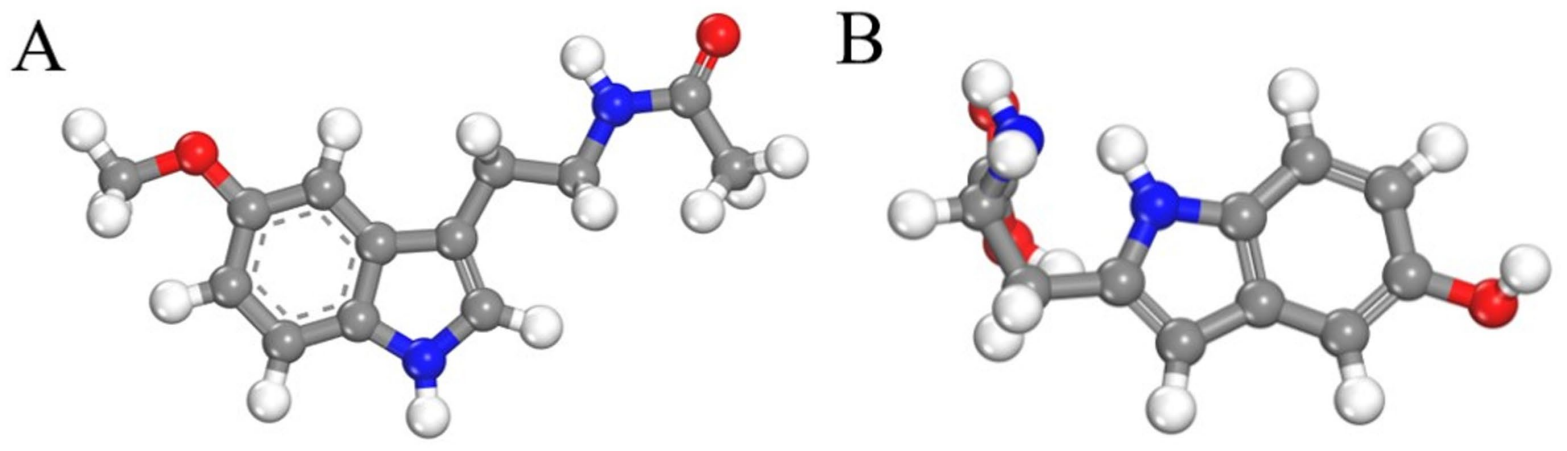
Molecular structures of melatonin **(A)** and 5-HTP **(B)**.

Melatonin (purity 98%, M813985, Shanghai, China) used in this study was purchased from McKinley Corporation, while 5-HTP (purity 99%, H136196, Shanghai, China) were acquired from Aladdin. Polyethylene (PE) powder (34–50 μm particle size, 434,272, America) purchased from Sigma-Aldrich. The molecular structures of melatonin and 5-HTP are depicted in Figure 1. PE was selected as the substrate for pure melatonin samples due to its low absorption characteristics in the THz band. All samples were used directly without further purification.

At room temperature, pure melatonin powder and 5-HTP were mixed separately with PE powder to prepare film samples of varying mass fractions. The total mass of each mixture was 150.0 g, with melatonin amounts of 37.5, 75.0, 112.5, and 150.0 g, corresponding to mass fractions of 25, 50, 75, and 100%, respectively.

L-tryptophan (purity 99%, T103480, Aladdin, Shanghai, China) mixtures were prepared by combining pure melatonin powder and 5-HTP with L-tryptophan in a 1:1 ratio, resulting in each mixture having a total mass of 150.0 mg.

Melatonin and 5-HTP dietary supplements were purchased from Tmall supermarket, with 150 mg of each sample being ground and compressed into tablets.

### 2.2 Raman, PXRD, and THz spectra testing systems

Raman spectroscopy is a widely used method for qualitative and quantitative analysis of substances, valued for its simplicity, non-destructiveness, and rapidity (26, 27).The experiment utilized a laser confocal Raman spectrometer produced by Renishaw Corporation (London, UK) with excitation by a 785 nm laser and measurement of data in the range of 150–3,500 cm^−1^.

PXRD is highly effective for studying crystal structures and rapid non-destructive detection (28, 29). The Smart Lab X-ray diffractometer from Rigaku Corporation (Tokyo, Japan) was employed in this study. Samples were placed on glass plates and data were collected within the range of $2\theta =20^{{}^{\circ}}-80^{{}^{\circ}}$ .

**Figure 2:**
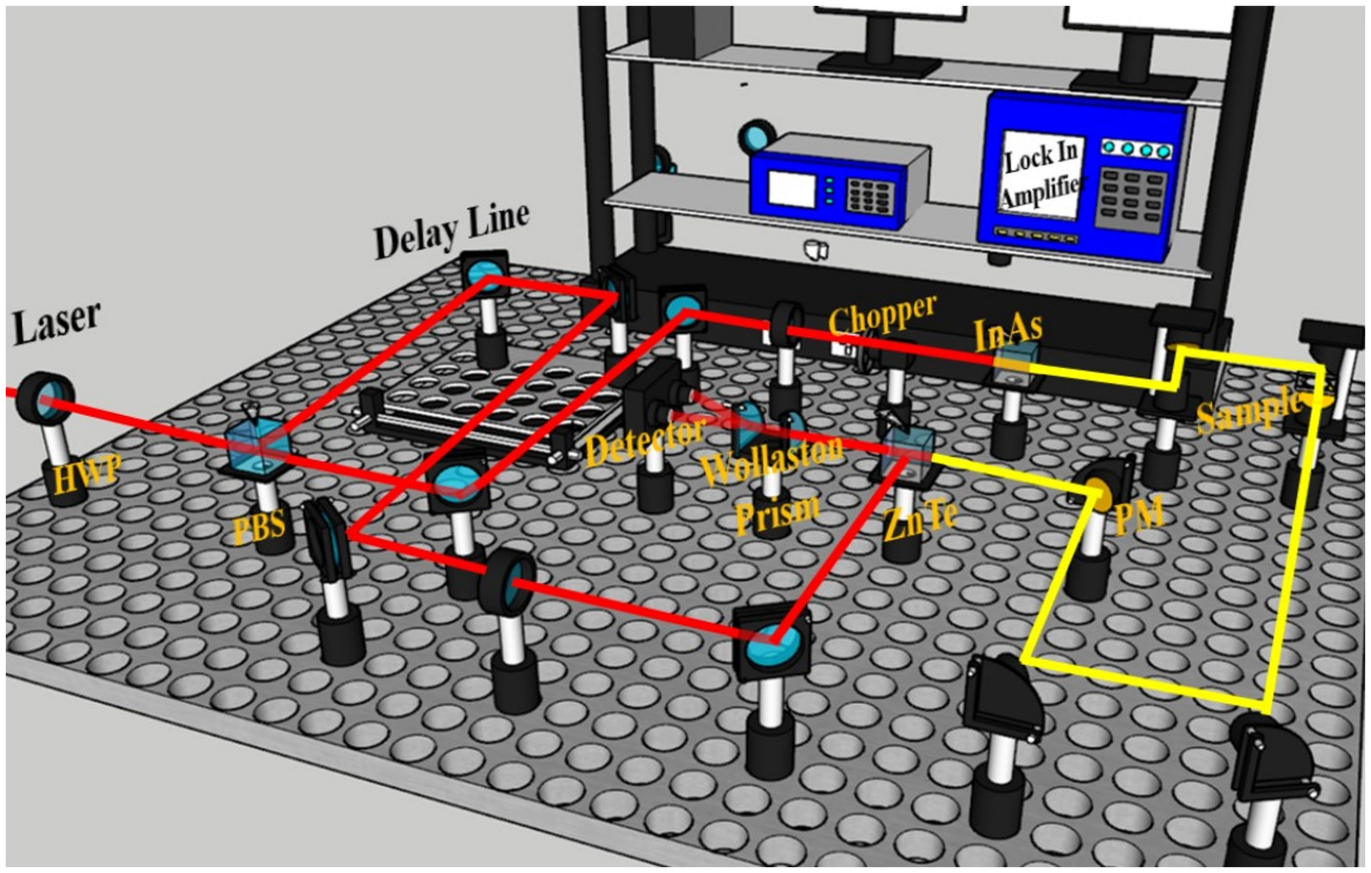
Schematic diagram of the THz-TDS system optical path.

A self-built THz-TDS system (Figure 2) was used for this research. The system comprises a femtosecond laser, THz radiation device, detection device, and time delay control system. In this setup, a femtosecond laser with a central wavelength of 800 nm, pulse width of 100 fs, repetition rate of 82 MHz and the output laser power is 3.4 W. The scanning step size is 10 μm, with a length of 3 cm. The frequency-domain resolution is 37 GHz, and the temporal resolution is 66 fs. The laser is split into pump and probe pulses after passing through a half-wave plate (HWP) and polarization beam splitter (PBS). The pump pulse excites an InAs crystal to generate THz pulses, which carry sample information through the sample placed between two off-axis parabolic mirrors to a ZnTe crystal. When THz waves irradiate the ZnTe crystal, its refractive index undergoes an-isotropic changes, altering the polarization state of the probe pulse. The signal is then collected via differential detection and amplified by a lock-in amplifier. Finally, data are processed and analyzed using a computer.

### 2.3 Experimental data processing

The THz-TDS spectra is converted to the frequency domain using Fourier transform. The optical parameters of the sample, including the complex transmission function H(*ω*), refractive index n(*ω*), and absorption coefficient *α*(*ω*), are calculated using Equations 1–3. (30, 31):


(1) $$H\left(\omega \right)=\frac{E_{sam}\left(\omega \right)}{E_{ref}\left(\omega \right)}=A\left(\omega \right)\exp\left(-i\Phi \left(\omega \right)\right)$$

(2) $$n\left(\omega \right)=\frac{\Phi \left(\omega \right)c}{\omega d}+1$$

(3) $$\alpha \left(\omega \right)=\frac{2}{d}\ln\left\{\frac{4n\left(\omega \right)}{A\left(\omega \right){\left[n\left(\omega \right)+1\right]}^{2}}\right\}$$

where $E_{sam}\left(\omega \right)$ and $E_{ref}\left(\omega \right)$ are the Fourier transform amplitudes of the sample ad reference signals, respectively. A(*ω*) is the ratio of the amplitudes of the sample signal to the reference signal, *Φ*(ω) is the phase difference, ω is the angular frequency, c is the speed of light in vacuum, d is the thickness of the sample.

### 2.4 Theoretical calculation

**Figure 3:**
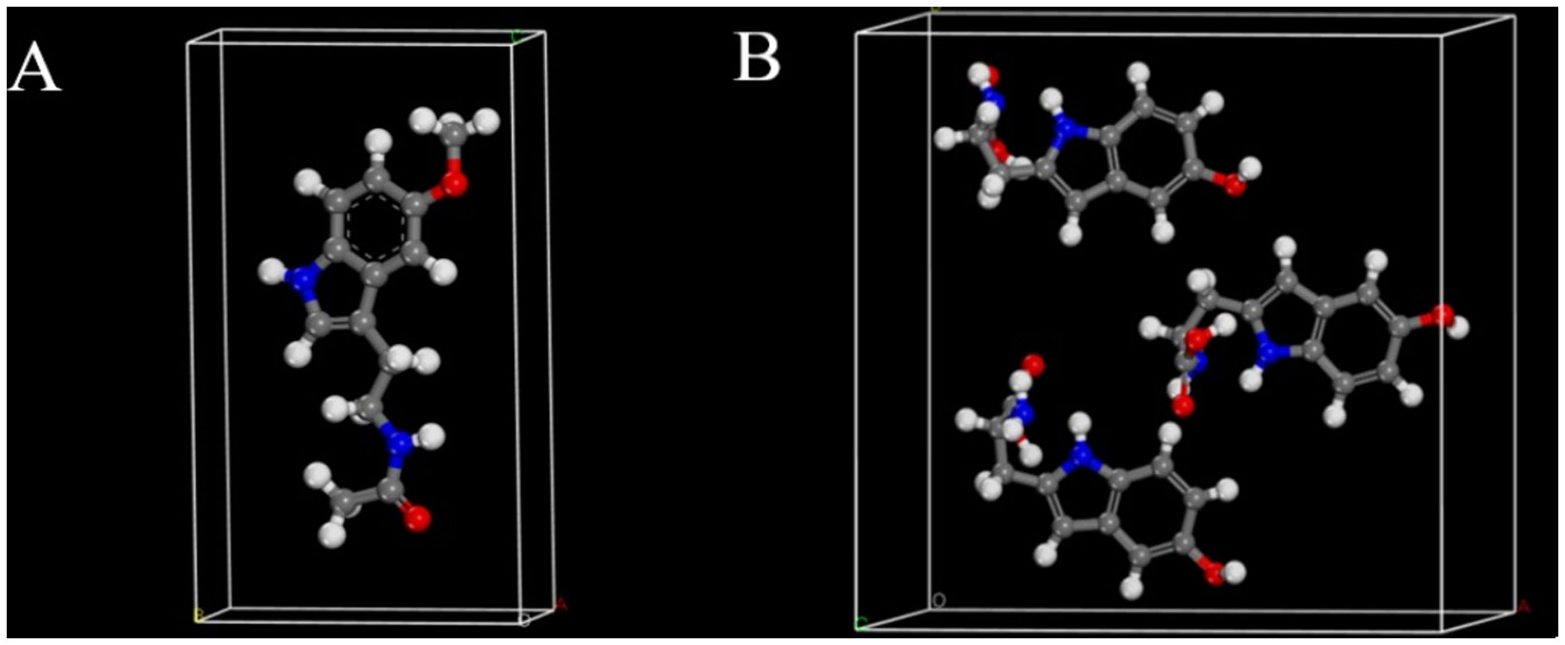
Cell structures of melatonin **(A)** and 5-HTP **(B)**.

In this study, the solid-state DFT calculations of melatonin and 5-HTP were con-ducted using the Cambridge Sequential Total Energy Package (CASTEP) within the Material Studio (MS) platform. Geometric optimization of crystal structures and vibrational spectra were calculated using the Perdew-Burke-Ernzerhof (PBE) ex-change-correlation potential and other parameters within the generalized gradient ap-proximation (GGA). The crystal cell structure of melatonin is constructed based on data from the Cambridge Crystallographic Data Center (CCDC), as shown in Figure 3.

## 3 Results and discussion

### 3.1 Raman spectroscopic analysis of melatonin and 5-HTP

**Figure 4:**
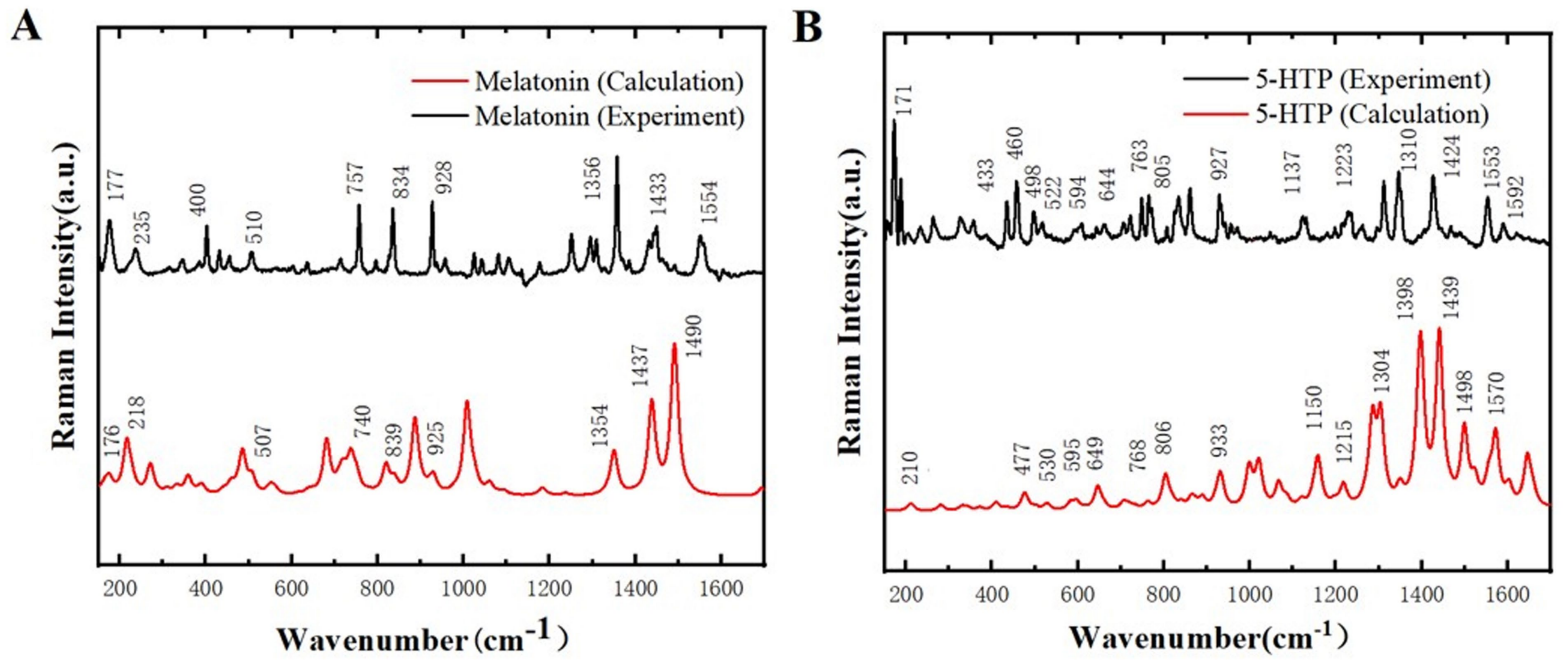
Comparison of Raman spectra between experimental results and simulated calculations. **(A)** Melatonin. **(B)** 5-HTP.

Raman spectroscopy is a technique that provides information about the chemical composition and structure of a sample by measuring the frequency shift of scattered light. Due to the sample does not require special treatment, it can maintain its original state, thereby minimizing the errors introduced during the sample preparation process in the experiment. Additionally, Raman spectroscopy is highly sensitive, capable of detecting even trace amounts of compounds in samples, which enables more accurate identification of chemical changes in samples. Figure 4 shows the Raman spectra of melatonin and 5-HTP between 170 and 1700 cm^−1^. It can be observed that melatonin and 5-HTP exhibit distinct characteristic peaks within this spectral range. Specifically, melatonin exhibits absorption peaks at 177, 235, 400, 510, 757, 834, 928, 1,356, 1,433, and 1,554 cm^−1^. The primary absorption peaks closely align with the simulated Raman spectra of melatonin, as illustrated in Figure 4A. On the other hand, 5-HTP exhibits absorption peaks at 171, 433, 460, 498, 522, 594, 644, 763, 805, 927, 1,137, 1,223, 1,310, 1,424, 1,553 and 1,592 cm^−1^, which also closely correspond to the simulated Raman spectrum of 5-HTP as shown in Figure 4B.

The position of Raman spectrum peak provides the information of specific chemical bonds or molecular vibrational modes in the sample, and the peak intensity reflects the content or concentration of these chemical bonds or molecular vibrational modes in the sample. MS simulation can only simulate the composition of chemical bonds and molecules in the sample, but cannot show the specific concentration of each chemical bond and molecule, which leads to different peak intensities. In this study, to prove the reliability of the sample, we only need to ensure that the peak positions of the simulation and experiment are basically the same, and do not need too much peak intensity. Therefore, it is reasonable to use this unit in the subsequent THz simulation. The difference in peak intensity is due to the difference in the content or con-centration of chemical bonds or molecular vibration modes in the crystal units used in the simulation compared with the experimental samples. For the slight difference in peak position, the reason may be that the simulation is carried out under ideal conditions, and the experiment cannot reach the ideal conditions, resulting in error (32, 33). At the same time, due to the existence of error, the two peaks may overlap when they are relatively close. Therefore, Raman spectroscopy indicates that the samples used in this study exhibit high purity and that no chemical changes occurred during the preparation process. This finding lays a solid foundation for further research on the THz spectral characteristics of the samples.

Raman spectroscopy has an obvious drawback in quantitative research. It often has a relatively high margin of error. There are several reasons for this. Firstly, the intensity of the Raman signal can be affected by various factors, such as the orientation of molecules in the sample, impurities that may cause interfering signals, and the optical properties of the sample container or the surrounding medium. These factors can lead to changes in the measured signal intensity, making it challenging to accurately quantify the analyte based solely on Raman spectroscopy. In addition, in Raman spectral analysis, when it comes to peak intensity measurement, there are various methods for baseline determination and a high degree of subjectivity. Different choices of fitting functions can also lead to differences in peak intensity values, which makes peak intensity measurement lack a unified and accurate standard. Therefore, when precise quantification of substances is required, relying solely on Raman spectroscopy may not produce the most accurate results, and additional calibration methods or complementary techniques may be needed to improve accuracy and reduce the margin of error.

### 3.2 PXRD analysis of melatonin and 5-HTP

**Figure 5:**
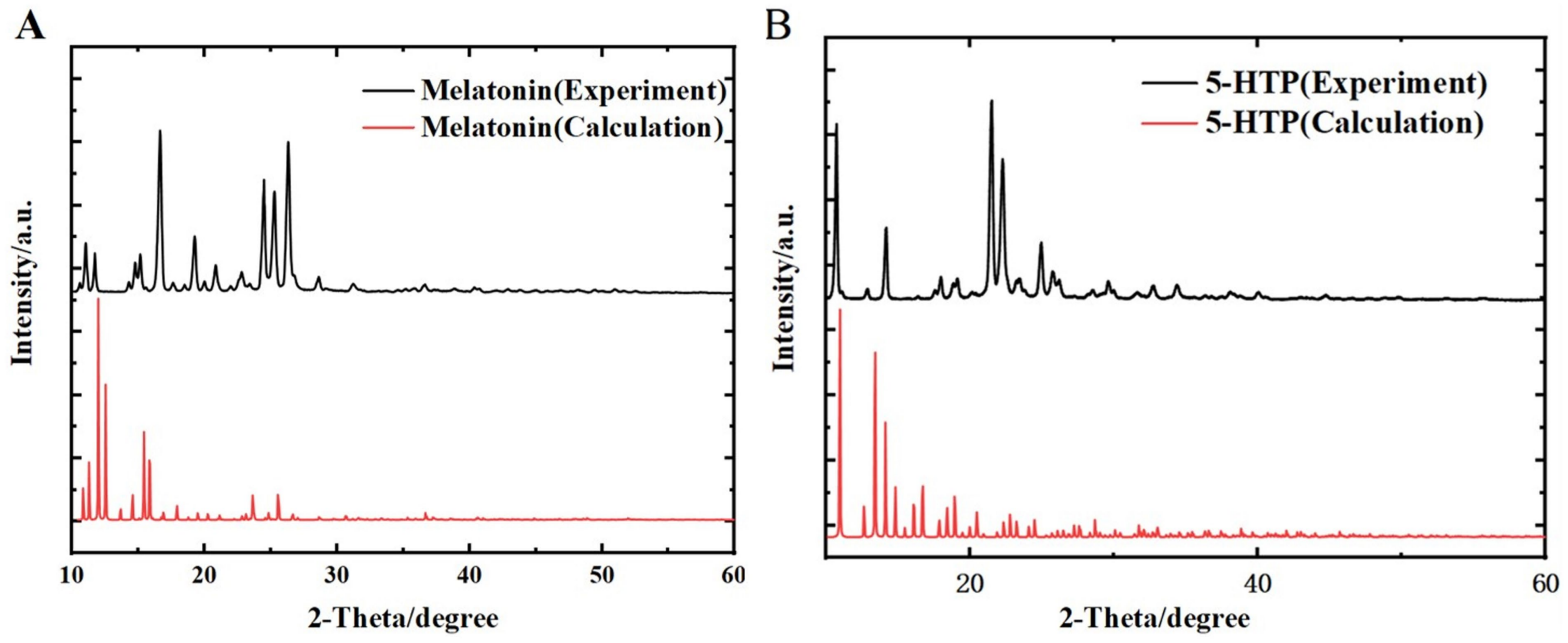
Comparison of PXRD spectra between experimental results and simulated calculations. **(A)** Melatonin. **(B)** 5-HTP.

To ensure the reliability of the melatonin and 5-HTP samples for THz simulation calculations and better analyze measurement data, we conducted PXRD to validate their basic structures. Figure 5A shows the PXRD patterns of melatonin obtained from both experimental and simulated data, with diffraction peaks matching well at 10.87°, 11.60°, 14.63°, 15.02°, 16.48°, 19.13°, 24.31°, 25.09°, and 26.17°. Figure 5B presents the PXRD patterns of 5-HTP obtained from experimental and simulated data, with diffraction peaks matching well at 10.59°, 14.05°, 21.37°, 22.15°, and 24.83°. This not only confirms the reliability of the structural simulations for melatonin and 5-HTP but also establishes a foundation for subsequent THz spectra studies of these substances.

### 3.3 THz characteristic analysis of melatonin and 5-HTP

**Figure 6:**
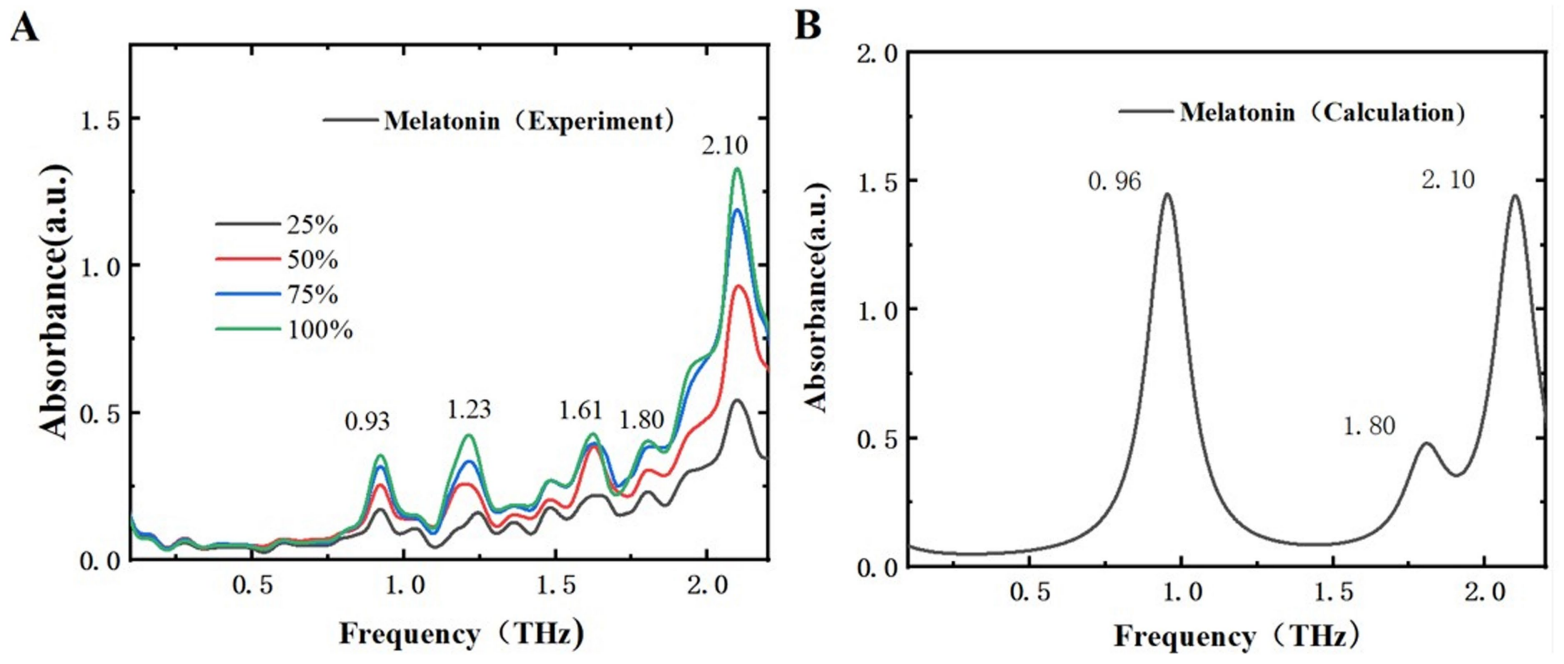
Comparison of absorption spectra of melatonin in different mass fractions. **(A)** THz experimental spectra. **(B)** THz simulated absorption spectra.

**Figure 7:**
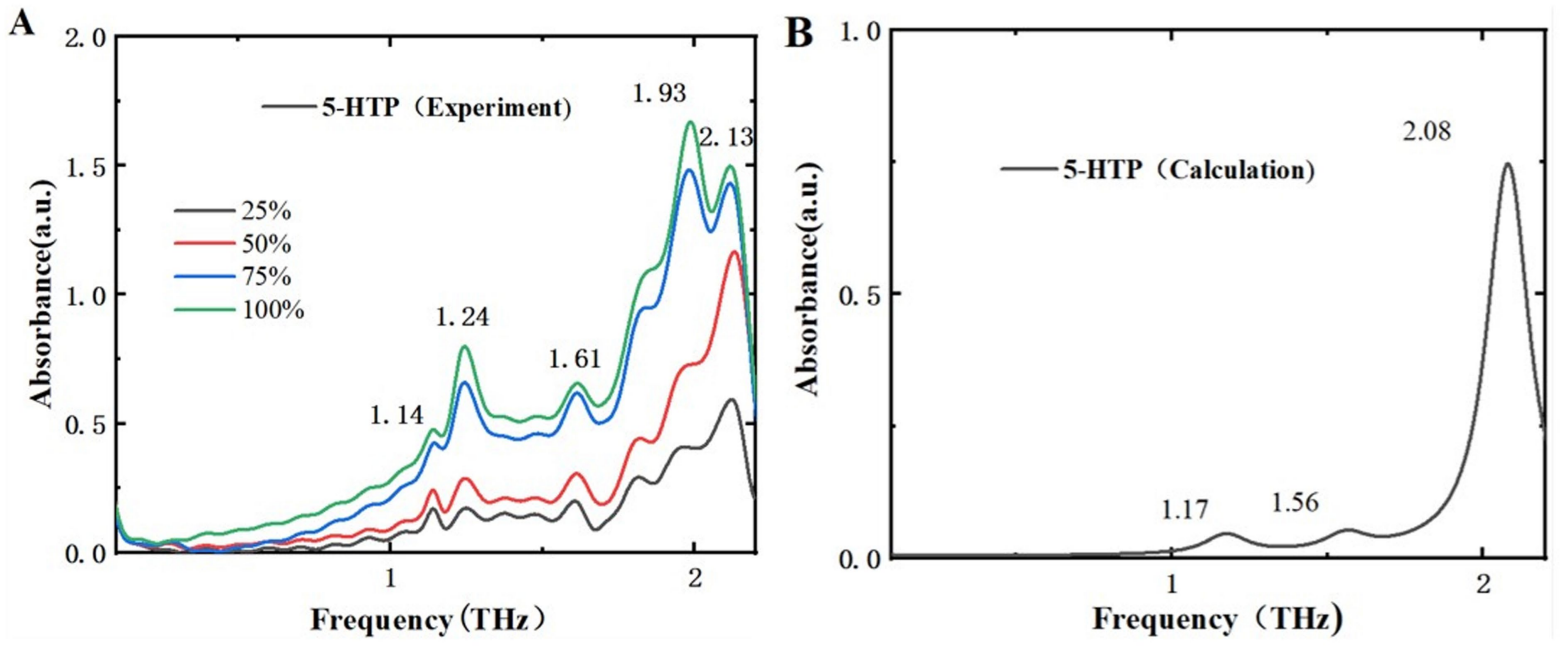
Comparison of absorption spectra of 5-HTP in different mass fractions. **(A)** THz experimental spectra. **(B)** THz simulated absorption spectra.

By comparing the THz time-domain and frequency-domain spectra of the samples, it is evident that the THz amplitude decreases with increasing sample mass fractions. To better differentiate these two samples, a detailed analysis of the THz absorption spectra for melatonin and 5-HTP was performed. The experimental and simulated THz ab-sorption spectra of melatonin and 5-HTP are shown in Figures 6, 7, respectively. Figure 6A illustrates the absorption spectra of melatonin at different mass fractions in THz experiments. The spectra reveal five distinct absorption peaks within the range of 0.1 to 2.2 THz, specifically at 0.93, 1.23, 1.61, 1.82, and 2.10 THz. This aligns closely with the peak positions of 0.93, 1.21, and 2.18 THz reported by Shen et al. using an air plasma THz-TDS system in the same frequency range (34). As for the newly observed peaks at 1.61 and 1.82 THz, we believe that this may be due to their large THz spectral range and low resolution in the low-frequency range, which hinders the clear characterization of characteristic peaks in the low-frequency region. Figure 6B presents the simulated THz absorption spectrum of melatonin. The peak at 2.10 THz corresponds perfectly with the experimental peak. However, due to the proximity of characteristic peaks, peaks such as those at 0.93 THz and 1.23 THz are combined into a single peak at 0.96 THz, and peaks at 1.61 THz and 1.82 THz are combined into a single peak at 1.80 THz in the simulation.

Figure 7A displays the THz absorption spectra of 5-HTP at different mass fractions, revealing 5 absorption peaks within the range of 0.1–2.2 THz at 1.14, 1.24, 1.61, 1.93, and 2.13 THz. Compared to Figure 7B, which shows the simulated THz absorption spectra of 5-HTP, the peak at 1.61 THz shifts to 1.56 THz. Additionally, the peaks at 1.14 THz and 1.24 THz are combined into a single peak at 1.17 THz, and the peaks at 1.93 THz and 2.13 THz are combined into a single peak at 2.08 THz. The simulation results are almost consistent with the experimental findings. Thus, we think that THz spectra can effectively distinguish between melatonin and 5-HTP.

The differences between experimental and simulated results can be attributed to several factors. Primarily, simulations frequently rely on ideal crystal structures that are difficult to precisely replicate in practical experimental measurements. Secondly, the absorption peaks of THz radiation are significantly affected by intermolecular interactions, particularly hydrogen bonding. These interactions might not be fully accounted for in simulation calculations, which can result in minor deviations in the observed absorption peaks. Furthermore, discrepancies between experimental and simulated spectra may also arise due to environmental differences.

**Figure 8:**
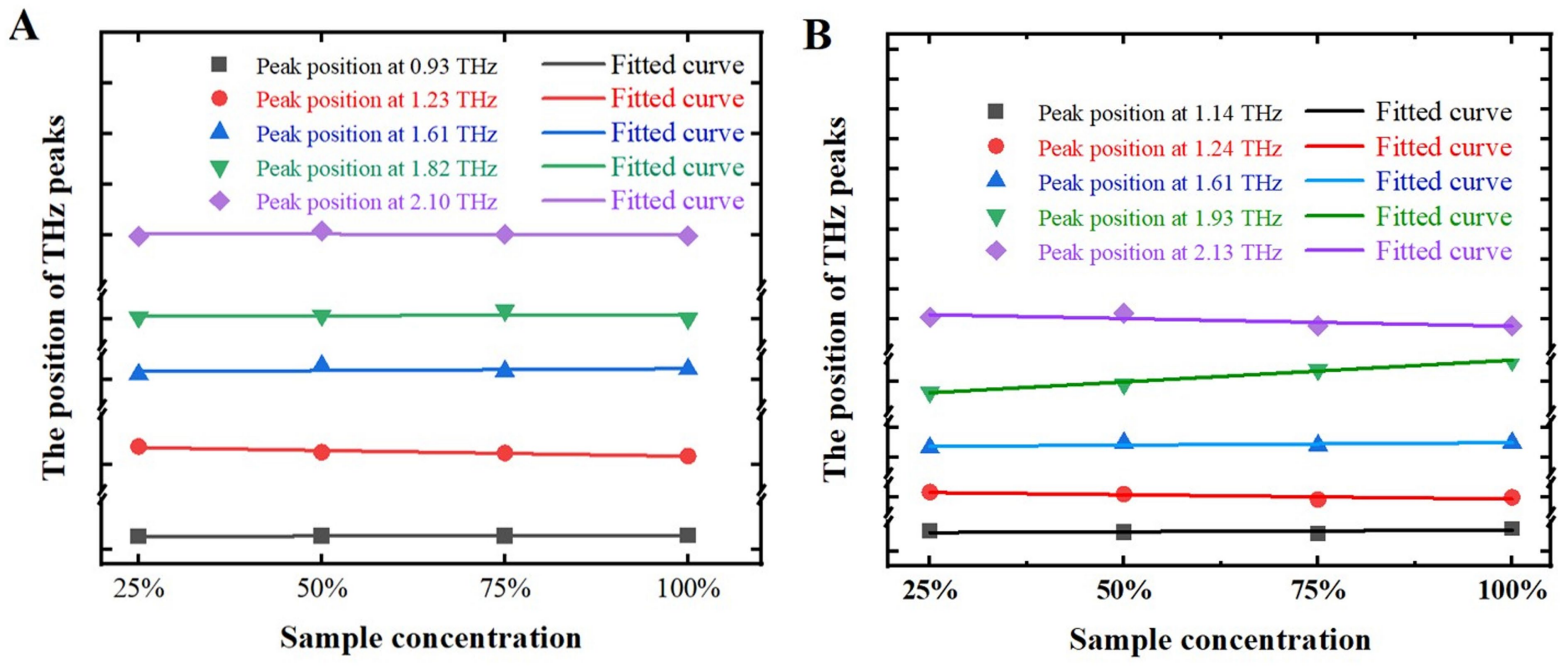
Positions of characteristic peaks at different mass fractions of samples. **(A)** Melatonin. **(B)** 5-HTP.

In order to more accurately investigate the relationship between the positions of THz characteristic peaks and the mass fractions of samples, and to more accurately determine the positions of these peaks, the positions of characteristic peaks at different mass fractions of the samples were studied, as shown in Figure 8. The x-axis represents sample mass fractions, while the y-axis indicates the positions of these characteristic peaks. Symbols such as square, circle, upward triangle, downward triangle, sideways triangle, and diamond denote the actual positions of THz characteristic peaks for both samples. Subsequently, we employed the least squares method to fit these characteristic peaks, resulting in corresponding fitting curves. The root means square errors (RMSE) for melatonin, as illustrated in Figure 8A, were 0.0013, 0.0128, 0.0014, 0.0042, 0.0008, and 0.0045, respectively. For 5-HTP, depicted in Figure 8B, the RMSE values were 0.0025, 0.0064, 0.0037, 0.0324, and 0.0177. Thus, it can be concluded that changes in mass fractions have minimal impact on the positions of characteristic peaks in the samples.

**Figure 9:**
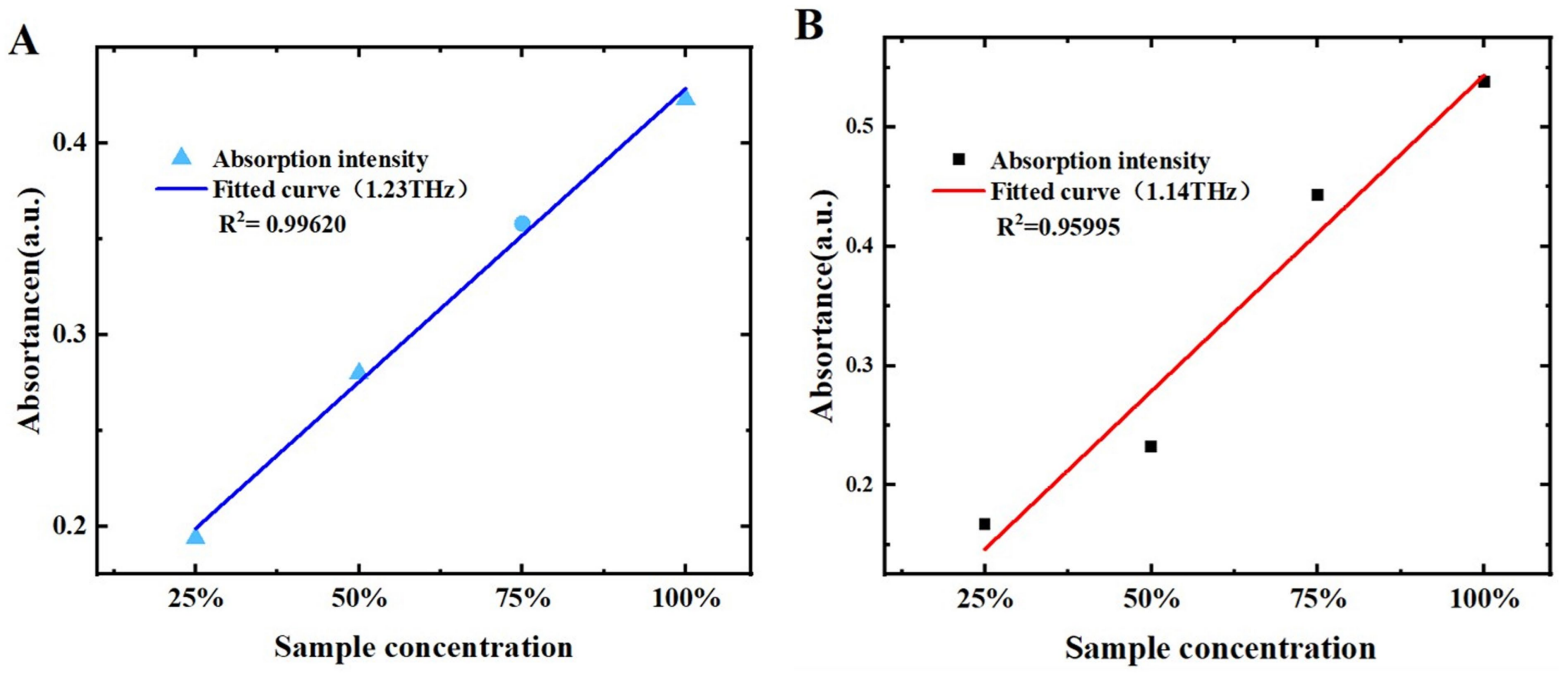
Relationship between different mass fractions of samples and absorption coefficients. **(A)** Melatonin at 1.23 THz. **(B)** 5-HTP at 1.14 THz.

To further qualitatively analyze melatonin and 5-HTP using THz spectra, the relationship between THz absorption coefficients and sample mass fractions was investigated. We performed linear fittings of melatonin and 5-HTP mass fractions with their THz ab-sorption coefficient intensities. The results of the melatonin fitting are shown in Table 1. The correlation coefficients at 0.93 THz, 1.23 THz, 1.61 THz, 1.82 THz, and 2.10 THz were 0.97601, 0.99620, 0.79779, 0.90777, and 0.90224, respectively. The relationship be-tween the mass fractions of melatonin and the peak at 1.23 THz exhibited the strongest correlation, as depicted in Figure 9A, where the x-axis and y-axis represent sample mass fractions and THz absorption coefficient intensity, respectively. This suggests that melatonin sample mass fractions can be predicted by measuring the peak at 1.23 THz. Table 2 shows the linear fitting results for 5-HTP. The correlation coefficients at 1.14 THz, 1.24 THz, 1.61 THz, 1.93 THz, and 2.13 THz were 0.95995, 0.95494, 0.90379, 0.95610, and 0.88475, respectively. The relationship between mass fractions and the peak at 1.14 THz showed the best fit, as shown in Figure 9B. It indicates that 5-HTP mass fractions can be predicted by measuring the peak at 1.14 THz.

**Table 1:** Linear fitting results of melatonin peak values with mass fractions.

Linear Fit: $y\left(x\right)=kx+b$			
x/THz	k	b	R^2^
0.93	0.0026	0.122	0.97601
1.23	0.0031	0.123	0.99620
1.61	0.0025	0.212	0.79779
1.82	0.0025	0.183	0.90777
2.10	0.0102	0.391	0.90224

**Table 2:** Linear fitting results of 5-HTP peak values with mass fractions.

Linear Fit: $y\left(x\right)=kx+b$			
x/THz	k	b	R^2^
1.14	0.0053	0.014	0.95995
1.24	0.0098	−0.092	0.95494
1.61	0.0068	0.049	0.90379
1.93	0.0194	−0.105	0.95610
2.13	0.0123	0.435	0.88475

These results demonstrate that THz spectra is a sensitive and quantifiable detection method, offering new possibilities for the development of dietary supplement testing.

### 3.4 THz characterization of L-tryptophan mixed with melatonin and 5-HTP, respectively

**Figure 10:**
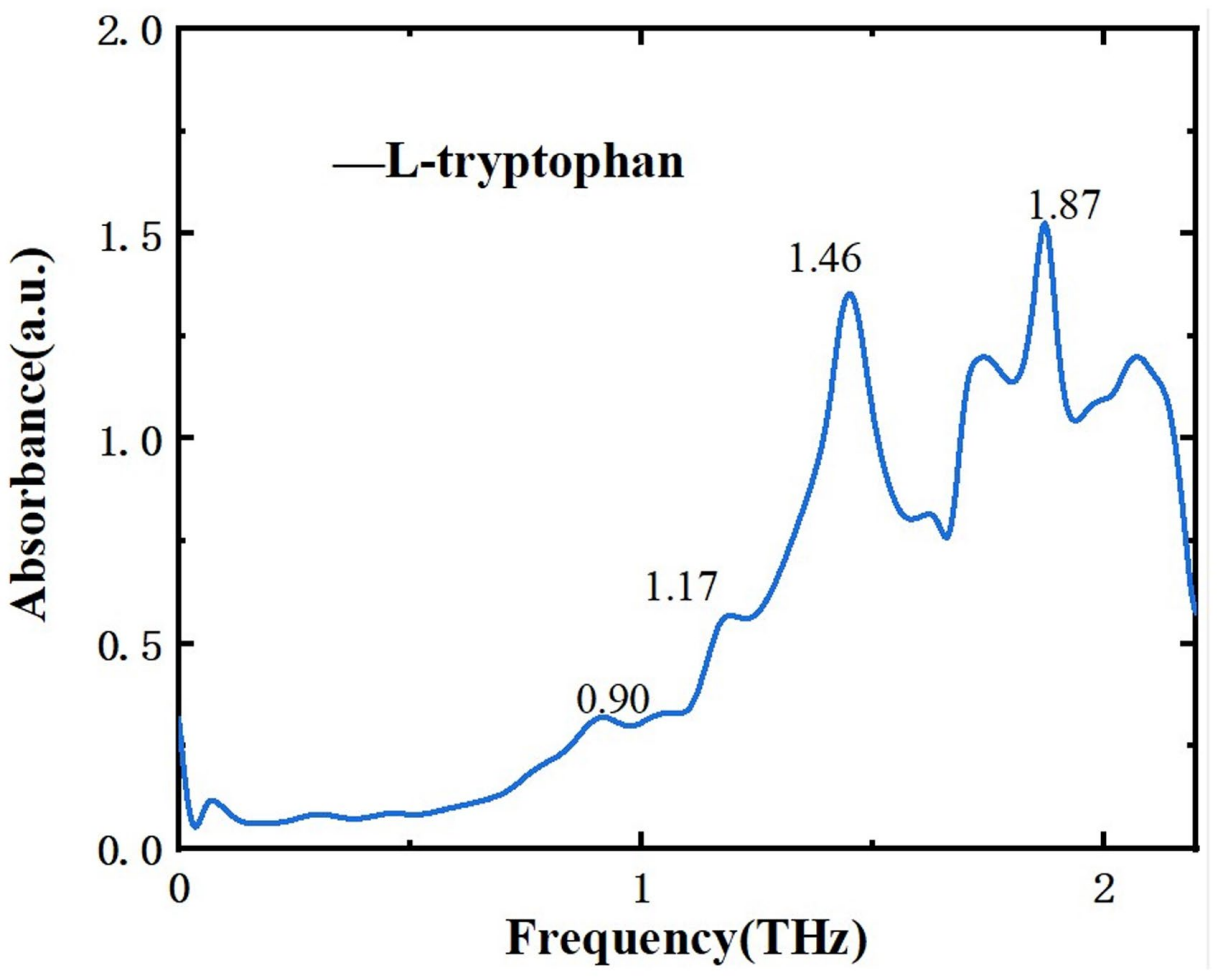
THz absorption spectra of L-tryptophan.

**Figure 11:**
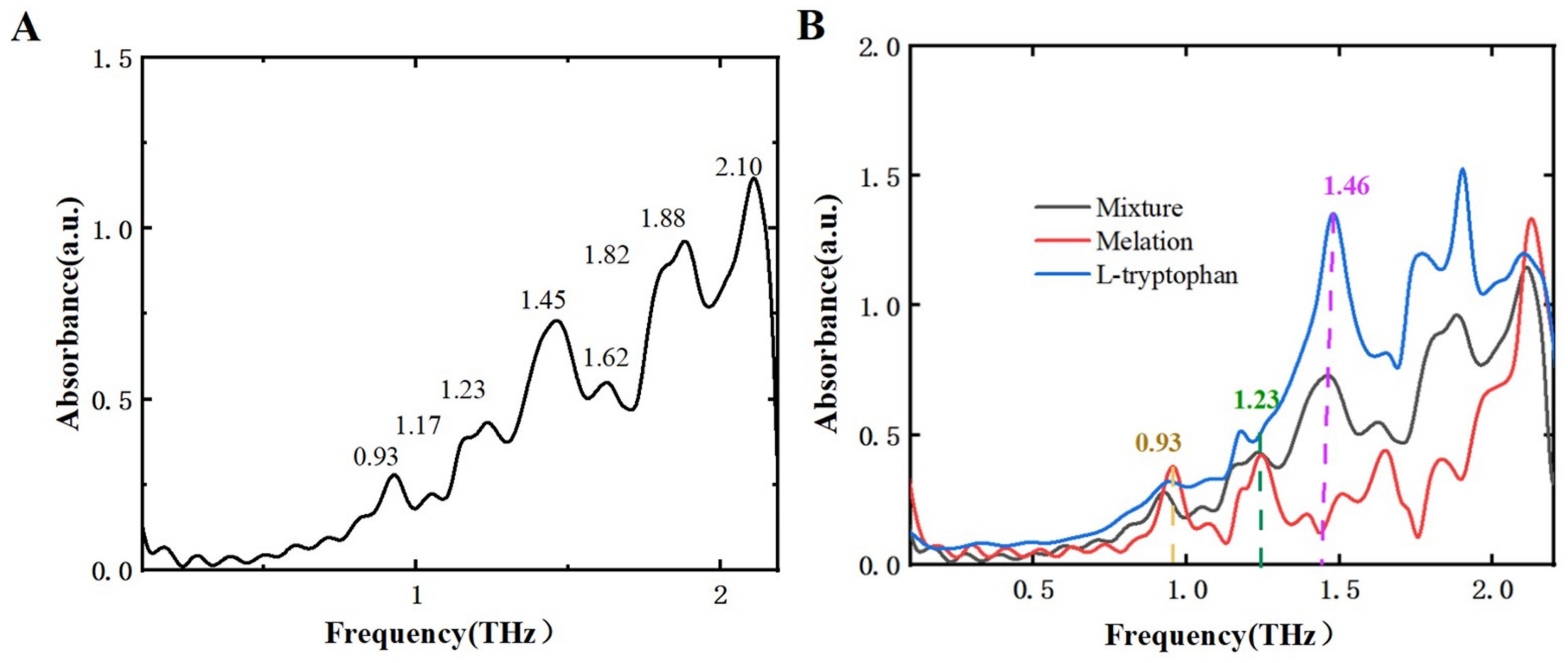
THz absorption spectra. **(A)** Mixture of L-tryptophan and melatonin. **(B)** Comparison of melatonin, L-tryptophan, and their mixture.

**Figure 12:**
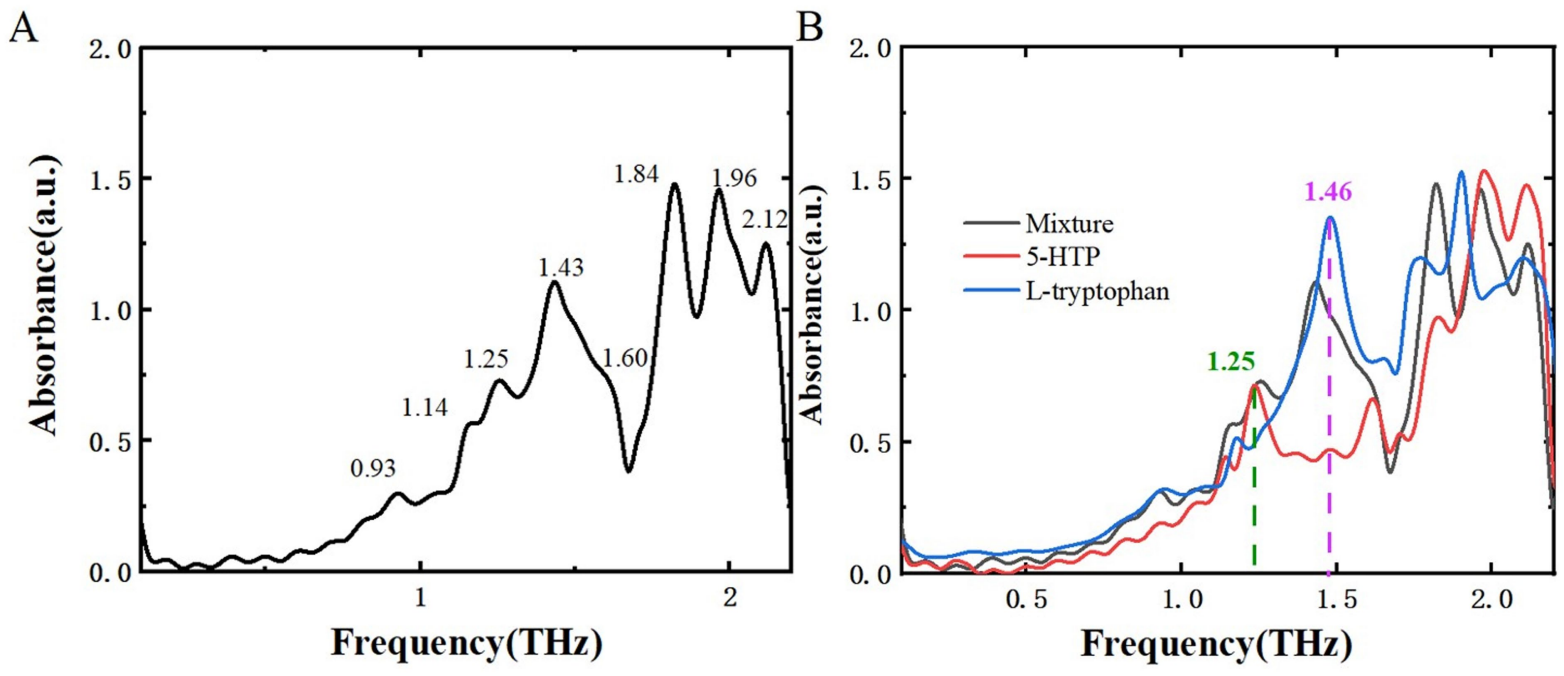
THz absorption spectra. **(A)** Mixture of L-tryptophan and 5-HTP. **(B)** Comparison of THz absorption spectra among 5-HTP, L-tryptophan, and their mixture.

After analyzing two samples using THz spectroscopy, research extended to L-tryptophan, a common byproduct generated during the production of melatonin and 5-HTP, and successfully identified it within the mixture. This additional analysis aims to reinforce food safety measures and broaden the potential applications of THz spectroscopy in pollutant detection. Figure 10 displays the THz absorption spectrum of L-tryptophan, showing characteristic peaks at 0.90, 1.17, 1.46, and 1.87 THz, consistent with reference (34, 35). Figure 11A illustrates the THz absorption spectrum of a mixture of L-tryptophan and melatonin, revealing eight absorption peaks at 0.93, 1.17, 1.23, 1.45, 1.62, 1.82, 1.88, and 2.10 THz, closely matching the absorption peaks of melatonin and L-tryptophan. Comparing the absorption spectra of melatonin, L-tryptophan, and their mixture in Figure 11B shows clear identification of L-tryptophan at 1.46 THz and identification of melatonin at 0.93 and 1.23 THz. Figure 12A is THz absorption spectrum of a mixture of L-tryptophan and 5-HTP, showing eight absorption peaks at 0.93, 1.14, 1.25, 1.43, 1.60, 1.84, 1.96, and 2.12 THz within the range of 0.1–2.2 THz, which align closely with the absorption peaks of 5-HTP and L-tryptophan. Figure 12B is comparison of the absorption spectra of 5-HTP, L-tryptophan, and their mixture. It can be observed that the mixture’s absorption spectrum identifies 5-HTP around 1.25 THz. Additionally, due to overlapping absorption peaks of 5-HTP and L-tryptophan between 1.8 THz and 2.2 THz, identification of L-tryptophan in the mixture primarily focuses on the lower frequency range, specifically around 1.46 THz. These results demonstrate that THz spectra can effectively distinguish common contaminants like L-tryptophan in mixtures, offering a new approach for identifying contaminants in dietary supplements.

### 3.5 THz characterization of melatonin and 5-HTP dietary supplements

**Figure 13:**
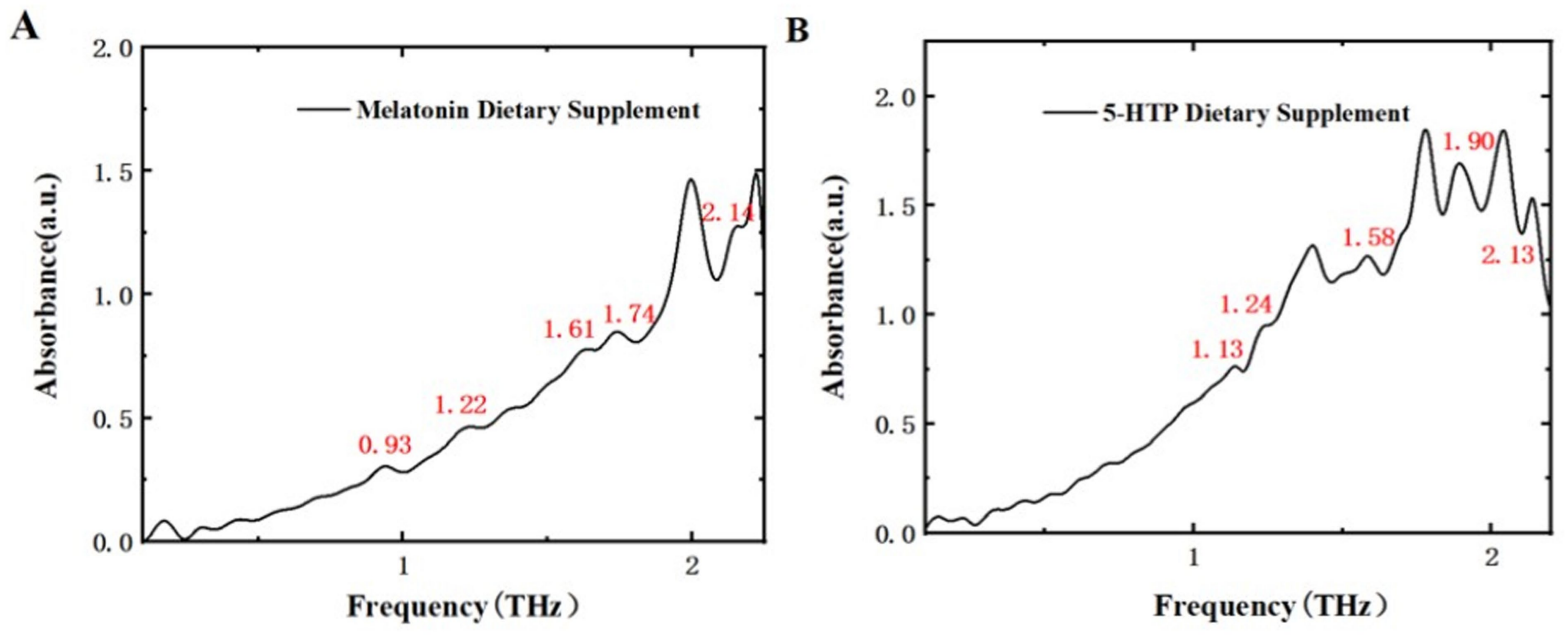
Dietary supplement THz absorption spectra. **(A)** Melatonin. **(B)** 5-HTP.

To evaluate the applicability of THz spectroscopy in commercial dietary supplements, two types of commercial dietary supplements were purchased from Tmall supermarket. These samples were ground, compressed, and directly subjected to THz detection. Figure 13A displays the THz absorption spectrum of the purchased melatonin dietary supplement. Compared to Figure 6A, the spectrum of the supplement shows characteristic peaks at positions similar to those of pure melatonin, specifically at 0.93, 1.22, 1.61, 1.74, and 2.14 THz. Figure 13B shows the THz absorption spectrum of the purchased 5-HTP dietary supplement. When compared with Figure 7A, this spectrum also exhibits characteristic peaks at positions similar to those of pure 5-HTP, at 1.13, 1.24, 1.58, 1.90, and 2.13 THz. These findings demonstrate that THz spectroscopy can effectively detect melatonin and 5-HTP in dietary supplements based on their characteristic peaks. This method offers a more convenient and efficient approach for market regulation of dietary supplements.

## 4 Conclusion

In this study, a step-by-step qualitative and quantitative analysis of melatonin and its precursor, 5-HTP, was conducted using Raman spectroscopy, PXRD, and THz spectroscopy technology. Raman spectroscopy was employed to verify the purity of the samples, ensuring that no chemical changes occurred during sample preparation. PXRD was utilized to confirm the crystal structure of the samples, thereby validating the accuracy of the DFT simulation calculations. Subsequently, THz-TDS experiments revealed characteristic peaks of melatonin and 5-HTP within the 0.1 to 2.2 THz range. The positions of these characteristic peaks showed no significant dependence on sample mass fractions, while their intensities exhibited a linear relationship with these fractions. Notably, the fittings at 1.23 THz and 1.14 THz yielded the best results, with correlation coefficients (R^2^) of 0.99620 and 0.95995, respectively. This establishes a solid foundation for the quantitative detection of dietary supplements using THz technology. Then, the detection of contaminants, especially L-tryptophan which might be present during the production of melatonin and 5-HTP dietary supplements, was investigated. Experimental results demonstrated that THz spectroscopy effectively distinguishes L-tryptophan in mixtures. Finally, commercially available dietary supplements were tested and it was found that the THz-TDS system could effectively detect melatonin and 5-HTP in these products. This demonstrates that THz spectroscopy can reliably identify key components in dietary supplements, providing a dependable method for monitoring and testing market products. These findings THz spectroscopy technology as a sensitive and quantifiable detection method, offering new possibilities for the development of dietary supplement testing.

## Data Availability

The raw data supporting the conclusions of this article will be made available by the authors, without undue reservation.

## References

[1] Favero G Franceschetti L Buffoli B Mohammed MH Reiter RJ Rodella LF. Melatonin: protection against age-related cardiac pathology. Ageing Res Rev. (2016) 35:336–49. doi: 10.1016/j.arr.2016.11.007, PMID: 27884595

[2] Shafabakhsh R Mirzaei H Asemi Z. Melatonin: A Promising Agent Targeting Leukemia. J Cell Biochem. (2019) 121:2730–8. doi: 10.1002/jcb.29495, PMID: 31713261

[3] Turner EH Loftis JM Blackwell AD. Serotonin a La carte: supplementation with the serotonin precursor 5-Hydroxytryptophan. Pharmacol Ther. (2006) 109:325–38. doi: 10.1016/j.pharmthera.2005.06.004, PMID: 16023217

[4] Israelyan N Colle AD Li Z Park Y Margolis KG. Effects of serotonin and slow-release 5-Htp on gastrointestinal motility in a mouse model of depression. Gastroenterology. (2019) 157. doi: 10.1053/j.gastro.2019.04.022 PMC6650329 31071306

[5] Meloni M Puligheddu M Carta M Cannas A Figorilli M Defazio G. Efficacy and safety of 5-Hydroxytryptophan on depression and apathy in Parkinson's disease: a preliminary finding. Eur J Neurol. (2020) 27:779–86. doi: 10.1111/ene.14179, PMID: 32067288

[6] Martin TG. Serotonin syndrome. Ann Emerg Med. (1996) 28:520–6. doi: 10.1016/S0196-0644(96)70116-6 8909274

[7] Dawson D van den Heuvel CJ. Integrating the actions of melatonin on human physiology. Ann Med. (2009) 30:95–102. doi: 10.3109/07853899808999390, PMID: 9556095

[8] Klarskov K Johnson KL Benson LM Gleich GJ Naylor S. Eosinophilia-myalgia syndrome case-associated contaminants in commercially available 5-Hydroxytryptophan. Adv Exp Med Biol. (1999) 467:461–8. doi: 10.1007/978-1-4615-4709-9_58, PMID: 10721089

[9] Williamson BL Tomlinson AJ Mishra PK Gleich GJ Naylor S. Structural characterization of contaminants found in commercial preparations of melatonin: similarities to case-related compounds from L-tryptophan associated with eosinophilia-myalgia syndrome. Chem Res Toxicol. (1998) 11:234–40. doi: 10.1021/tx970202h, PMID: 9544622

[10] Klarskov K Gagnon H Boudreault P-L Normandin C Plancq B Marsault E. Structure determination of disease associated peak Aaa from L-tryptophan implicated in the eosinophilia-myalgia syndrome. Toxicol Lett. (2018) 282:71–80. doi: 10.1016/j.toxlet.2017.10.012, PMID: 29037509

[11] Francescangeli J Karamchandani K Powell M Bonavia A. The serotonin syndrome: from molecular mechanisms to clinical practice. Int J Mol Sci. (2019) 20:2288. doi: 10.3390/ijms20092288, PMID: 31075831 PMC6539562

[12] Cerezo AB Leal N Lvarez-Fernández MA Hornedo-Ortega R García-Parrilla MC. Quality control and determination of melatonin in food supplements. J Food Compos Anal. (2015) 45:80–6. doi: 10.1016/j.jfca.2015.09.013, PMID: 39812234

[13] Padumanonda T Johns J Sangkasat A Tiyaworanant S. Determination of melatonin content in traditional Thai herbal remedies used as sleeping aids. Daru-J Faculty of Pharmacy. (2014) 22:6. doi: 10.1186/2008-2231-22-6 PMC3913336 24393215

[14] Yakupova Z Yakubenko A Bogdanova P Godunov P Vakh C Garmonov S. Solidified floating organic drop microextraction procedure based on deep eutectic solvent for the determination of melatonin in pharmaceuticals and dietary supplements. Microchem J. (2023) 187:108373. doi: 10.1016/j.microc.2022.108373

[15] Alpar N Pınar PT Yardım Y Şentürk Z. Voltammetric method for the simultaneous determination of melatonin and pyridoxine in dietary supplements using a Cathodically pretreated boron‐doped diamond electrode. Electroanalysis. (2017) 29:1691–9. doi: 10.1002/elan.201700077

[16] Li MD Tseng WL Cheng TL. Ultrasensitive detection of Indoleamines by combination of nanoparticle-based extraction with capillary electrophoresis/laser-induced native fluorescence. J Chromatogr A. (2009) 1216:6451–8. doi: 10.1016/j.chroma.2009.07.034, PMID: 19646710

[17] Li W Wu X Yuan X Zhou W. Rapid evaluation of Γ-aminobutyric acid in foodstuffs by direct real-time mass spectrometry. Food Chem. (2019) 277:617–23. doi: 10.1016/j.foodchem.2018.10.127, PMID: 30502194

[18] Shao Y Gu W Qiu Y Wang S Zhuang S. Lipids monitoring in Scenedesmus Obliquus based on terahertz technology. Biotechnol Biofuels. (2020) 13:161. doi: 10.1186/s13068-020-01801-0, PMID: 32944077 PMC7493189

[19] Cherkasova OP Serdyukov DS Ratushnyak AS Nemova EF Tuchin VV. Effects of terahertz radiation on living cells: a review. Opt Spectrosc. (2020) 128:855–66. doi: 10.1134/S0030400X20060041

[20] Afsah〩ejri L Hajeb P Ara P Ehsani RJ. A comprehensive review on food applications of terahertz spectroscopy and imaging. Compr Rev Food Sci Food Saf. (2019) 3. doi: 10.1111/1541-4337.12490 33336912

[21] Baek SH Kang JH Hwang YH Ok KM Kwak K Chun HS. Detection of Methomyl, a carbamate insecticide, in food matrices using terahertz time-domain spectroscopy. J Infrared Millim Terahertz Waves. (2015) 37:486–97. doi: 10.1007/s10762-015-0234-9, PMID: 39808227

[22] Wang Y Zhao Z Qin J Liu H Liu A Xu M. Rapid in situ analysis of L-histidine and alpha-lactose in dietary supplements by fingerprint peaks using terahertz frequency-domain spectroscopy. Talanta: the international journal of pure and applied. Anal Chem. (2020) 208:120469. doi: 10.1016/j.talanta.2019.120469, PMID: 31816746

[23] Nazarov MM Cherkasova OP Shkurinov AP. A comprehensive study of albumin solutions in the extended terahertz frequency range. J Infrared Millim Terahertz Waves. (2018) 39:840–53. doi: 10.1007/s10762-018-0513-3

[24] Han X Yan S Zang Z Wei D Du C. Label-free protein detection using terahertz time-domain spectroscopy. Biomed Opt Express. (2018) 9:994–1005. doi: 10.1364/BOE.9.000994, PMID: 29541499 PMC5846544

[25] Dragoman D Dragoman M. Terahertz fields and applications. Progress in Quantum Electronics. (2004) 28:1–66. doi: 10.1016/S0079-6727(03)00058-2

[26] Pirutin SK Jia S Yusipovich AI Shank MA Parshina EY Rubin AB. Vibrational spectroscopy as a tool for bioanalytical and biomonitoring studies. Int J Mol Sci. (2023) 24:6947. doi: 10.3390/ijms24086947, PMID: 37108111 PMC10138916

[27] Hao R Zhao J Liu J You H Fang J. Remote Raman detection of trace explosives by laser beam focusing and Plasmonic spray enhancement methods. Anal Chem. (2022) 94:11230–7. doi: 10.1021/acs.analchem.2c01732, PMID: 35921536

[28] Paranthaman R Moses JA Anandharamakrishnan C. Powder X-ray diffraction conditions for screening curcumin in turmeric powder. J Food Meas Charact. (2022) 16:1105–13. doi: 10.1007/s11694-021-01225-w

[29] Paranthaman R Moses JA Anandharamakrishnan C. Development and validation of a screening method for simultaneous detection of Kbro3 and Kio3 in baking ingredients and additives using powder Xrd. J Food Compos Anal. (2021) 102:104007. doi: 10.1016/j.jfca.2021.104007

[30] Dorney TD Baraniuk RG Mittleman DM. Material parameter estimation with terahertz time-domain spectroscopy. J Optical Society of America A Optics Image Sci Vision. (2001) 18:1562–71. doi: 10.1364/JOSAA.18.001562, PMID: 11444549

[31] Duvillaret L Garet F Coutaz JL. A reliable method for extraction of material parameters in terahertz time-domain spectroscopy. IEEE J Selected Topics in Quantum Electronics. (2002) 2:739–46. doi: 10.1109/2944.571775, PMID: 39573497

[32] Zong S Ren GH Li S Zhang B Zhang J Qi W. Terahertz time-domain spectroscopy of L-histidine hydrochloride monohydrate. J Mol Struct. (2018):S0022286017317088. doi: 10.1016/j.molstruc.2017.12.088

[33] Fang J Zhang Z Bo Y Xue J Du Y. Vibrational spectral and structural characterization of multicomponent ternary co-crystal formation within acetazolamide, nicotinamide and 2-Pyridone. Spectrochimica Acta Part A Molecular and Biomolecular Spectroscopy. (2020) 245:118885. doi: 10.1016/j.saa.2020.118885, PMID: 32920445

[34] Shen J Zhu Z Zhang Z Guo C Zhao H. Ultra-broadband terahertz fingerprint Spectrum of melatonin with vibrational mode analysis. Spectrochimica Acta Part A Molecular and Biomolecular Spectroscopy. (2021) 247:119141. doi: 10.1016/j.saa.2020.119141, PMID: 33188973

[35] Yi WT Yu JP Xu YT Wang F Yu Q Sun HJ. Broadband terahertz spectroscopy of amino acids. Instrum Sci Technol. (2017) 45:423–39. doi: 10.1080/10739149.2016.1270961

